# The features of the coastal fronts in the Eastern Guangdong coastal waters during the downwelling-favorable wind period

**DOI:** 10.1038/s41598-021-89649-8

**Published:** 2021-05-13

**Authors:** Chaoyu Yang, Haibin Ye

**Affiliations:** 1 South China Sea Marine Prediction Center, State Oceanic Administration, Guangzhou, China; 2 Key Laboratory of Marine Environmental Survey Technology and Application, Ministry of Natural Resources, MNR, Guangzhou, China; 3 Guangdong Provincial Key Laboratory of Marine Resources and Coastal Engineering, Guangzhou, China; 4 State Key Laboratory of Tropical Oceanography, South China Sea Institute of Oceanology, Chinese Academy of Sciences, No. 164, West Xin Gang Road, Guangzhou, 510301 China; 5 Southern Marine Science and Engineering Guangdong Laboratory (Guangzhou), Guangzhou, China

**Keywords:** Physical oceanography, Environmental impact

## Abstract

A coastal front was detected in the eastern Guangdong (EGD) coastal waters during a downwelling-favorable wind period by using the diffuse attenuation coefficient at 490 nm (*K*_*d*_(490)). Long-term satellite data, meteorological data and hydrographic data collected from 2003 to 2017 were jointly utilized to analyze the environmental factors affecting coastal fronts. The intensities of the coastal fronts were found to be associated with the downwelling intensity. The monthly mean *K*_*d*_(490) anomalies in shallow coastal waters less than 25 m deep along the EGD coast and the monthly mean Ekman pumping velocities retrieved by the ERA5 dataset were negatively correlated, with a Pearson correlation of − 0.71. The fronts started in October, became weaker and gradually disappeared after January, extending southwestward from the southeastern coast of Guangdong Province to the Wanshan Archipelago in the South China Sea (SCS). The cross-frontal differences in the mean *K*_*d*_(490) values could reach 3.7 m^−1^. Noticeable peaks were found in the meridional distribution of the mean *K*_*d*_(490) values at 22.5°N and 22.2°N and in the zonal distribution of the mean *K*_*d*_(490) values at 114.7°E and 114.4°E. The peaks tended to narrow as the latitude increased. The average coastal surface currents obtained from the global Hybrid Coordinate Ocean Model (HYCOM) showed that waters with high nutrient and sediment contents in the Fujian and Zhejiang coastal areas in the southern part of the East China Sea could flow into the SCS. The directions and lengths of the fronts were found to be associated with the flow advection.

## Introduction

Coastal fronts are principal hydrographic features that usually occur in regions where the velocity, density, temperature, or salinity gradient is extremely high or a significant discontinuity exists^1,2^. Coastal fronts could have substantial effects on pollutant drift (such as oil slicks), algal blooms and larval distributions^3,4^. Ocean foam, detritus, surface films, fish and oil slicks have been found to be concentrated at fronts^5^. Previous researchers have illustrated that coastal fronts can produce marked surface convergence conditions. Phytoplankton chlorophyll has also been observed along these convergence zones. Coastal fronts have been found to increase the productivity of zooplankton^6^. The combined use of high-resolution Earth observations and numerical models has enabled the identification of dynamic regions at small scales and sub-mesoscales for analyses of coastal front structures and their interactions^7^. Coastal fronts play important roles in dynamic and chemical processes.

The South China Sea (SCS) is influenced by the East Asian monsoon system, which is characterized by prevailing northeasterly winds in winter and southwesterly winds in summer^8^. In the SCS, ocean circulations are mainly driven by strong northeasterly winds that prevail in winter and by weak southwesterly winds that prevail in summer^9,10^. Over the last few decades, oceanographic studies on the SCS have been centered on summer upwelling phenomena and the interactions between the Pearl River plume and the coastal currents^11–13^. However, little research has been conducted on the coastal fronts that occur during periods of strong downwelling-favorable winds occurring in the northern South China Sea (NSCS) in winter. Downwelling is one of the most significant dynamic phenomena in the NSCS. It has a great influence on the nearshore circulation along the eastern Guangdong (EGD) coast. The EGD coastal fronts that occur in the EGD coastal waters not only have a pronounced influence on the hydrologic characteristics of the region but are also important for algal blooms, pollutant dispersion, seafloor sediments, and ship navigation in the NSCS. However, the features of coastal fronts in EGD coastal waters have not yet been investigated extensively. With the development of remote sensing techniques, satellite data have become an effective data source for studying the frequency and region of coastal front occurrences in EGD coastal waters.

The purpose of the study is to analyze the locations, occurrence times, durations, intensities and convergence properties of coastal fronts in the EGD coastal region by applying remote sensing techniques. In this study, ocean color data and meteorological and hydrographic data were jointly utilized to illustrate the most important factors influencing the coastal fronts.

## Materials and method

### Study area

**Figure 1:**
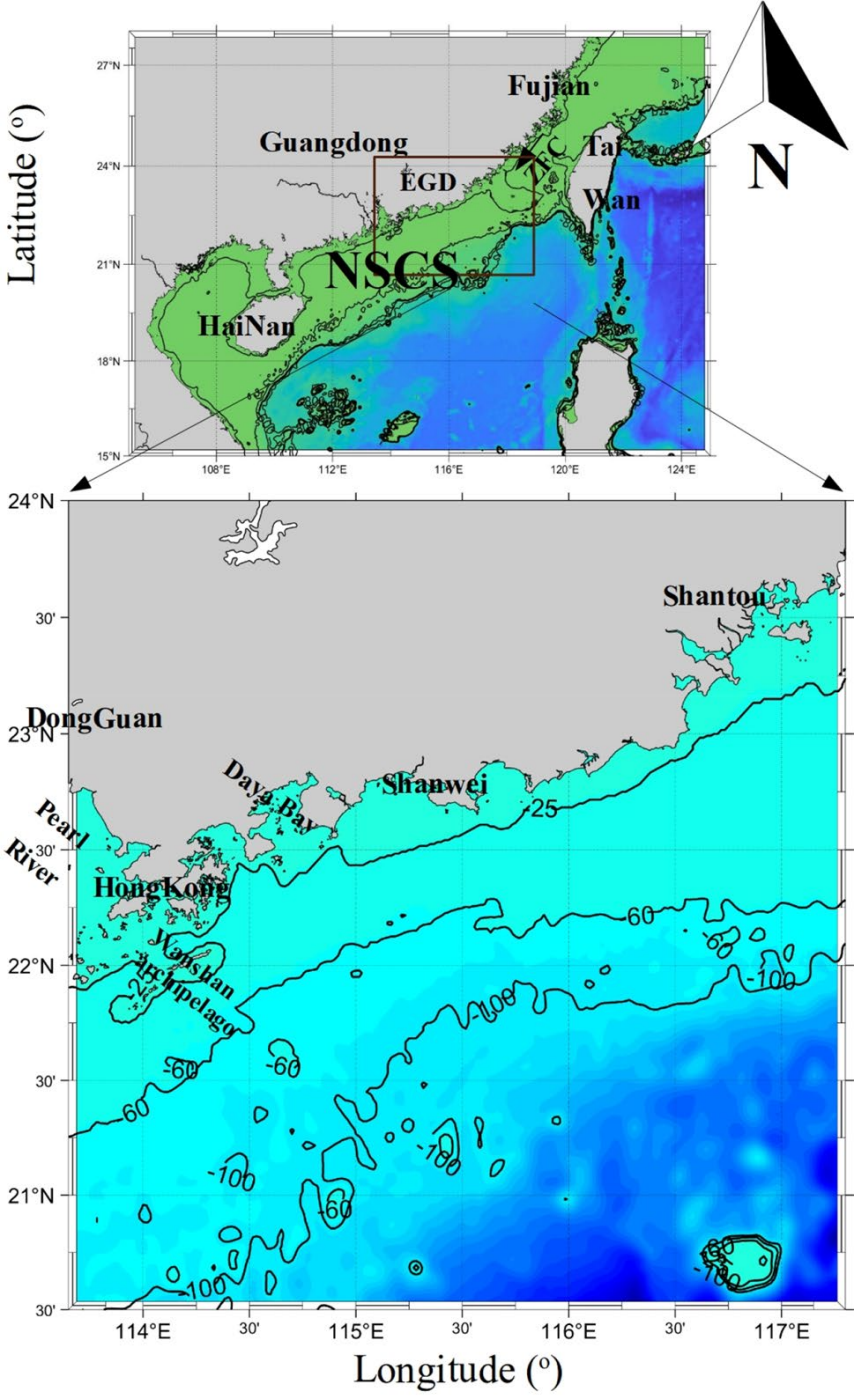
Map of the study area. The program R (version 3.6.3, available at https://www.r-project.org) was applied for the map.

EGD coastal waters are known as subtropical coastal regions with high biological productivity and are located in the NSCS^14^. The EGD coastal area (Fig. 1) is characterized by a complicated hydrodynamic system driven by many physical factors, including river discharge, bottom topography, monsoon winds, and coastal currents^15^. The region is influenced by the East Asian monsoon system, which is characterized by prevailing northeasterly and southwesterly winds in winter and summer, respectively^8^. In this study, the seasons refer to those of the Northern Hemisphere, for example, autumn refers to September, October, and November. With increasing human activity, EGD coastal waters have been contaminated by industrial pollution, agricultural runoff, and domestic sewage^16^.

### Ancillary data

Level-1B moderate-resolution imaging spectroradiometer (MODIS) Aqua imagery from 2003 to 2017 were obtained from the online archive of the Level-1 Atmosphere Archive & Distribution System (LAADS) Distributed Active Archive Center (DAAC). The SeaWiFS data analysis system (SeaDAS, version 7.5.1) was applied to process the data. For the data processing procedure, an iterative f/Q BRDF correction and MUMM-based atmospheric correction were chosen^17–19^. In addition, sea surface temperature (SST) products at a 1-km resolution (Level 2) were obtained from the Physical Oceanography Distributed Active Data Center (PO. DAAC) of the NASA Jet Propulsion Laboratory; these data were derived by the NASA Ocean Biology Processing Group (OBPG) and can be accessed at the following site: https://podaac.jpl.nasa.gov.

The meteorological data were obtained from the latest climate reanalysis ERA5 product released by the European Centre for Medium-Range Weather Forecasts (ECMWF, https://www.ecmwf.int/en/research/climate-reanalysis). ERA5 was carried out by using the ECMWFS' Earth System model IFS, cycle 41r2. The ERA5 data used in this study, which are on regular latitude–longitude grids at a 0.25° × 0.25° resolution with atmospheric parameters at 37 pressure levels, are available in the climate data store (https://climate.copernicus.eu/climate-reanalysis) and cover the period from 1950 to the present. The hourly wind speeds at a height of 10 m above the sea surface were extracted from the MODIS pixels in EGD for the 2003–2017 period.

The hydrological data were acquired from the global Hybrid Coordinate Ocean Model (HYCOM) analysis dataset. The HYCOM-Navy Coupled Ocean Data Assimilation (HYCOM + NCODA) Global (1/12)° Analysis product was provided by the Global Ocean Forecasting System (GOFS). The data assimilation scheme is a three-dimensional variational scheme (3DVAR)^20^. Three-hourly velocity fields obtained in 2005 were used in this study.

ETOPO1 bathymetry grids at a 1-arcminute resolution were obtained from the National Centers for Environmental Information and applied in this study (NCEI, https://www.ngdc.noaa.gov/mgg/global/global.html). The bathymetric grids were extracted to the same grid resolution as that of the MODIS imagery.

### Ancillary software

To process the MODIS data, the SeaWiFS data analysis system (SeaDAS, version 7.5.1, available at https://seadas.gsfc.nasa.gov/) developed by NASA was applied for image preprocessing. Several atmospheric correction algorithms exist, including Bailey’s et al.^21^ iterative correction model, which was used to remove nonnegligible NIR radiance from the NIR signals prior to aerosol determination, Wang and Shi’s^22^ model, and the Management Unit of the North Seas Mathematical Models (MUMM)^23^ (more details can be found at https://oceancolor.gsfc.nasa.gov/seadas/). In this study, the MUMM model was applied. The L1b data were processed to obtain the Rayleigh scattering corrected reflectance values by applying the default atmospheric correction algorithm during the first step. The ETOPO1 bathymetry grids were extracted to the same grid as the MODIS imagery. Then, the Rayleigh scattering corrected reflectance values were extracted over the clear water surface (a region with a water depth greater than 200 m). A regression line was fitted to the Rayleigh scattering corrected reflectance values at 748 nm and 869 nm. The slope of the regression line between the two bands was applied to determine epsilon, which is an input of the MUMM model used to correct for the aerosol scattering contribution. Moreover, the program R (version 3.6.3, available at https://www.r-project.org) and C Programming were applied for the statistical computing and creation of graphics.

### Method

#### Model for *K*_*d*_(490) retrieval

In aquatic systems, the light diffuse attenuation coefficient *K*_*d*_(*λ*) is defined by the exponential decrease in irradiance that occurs with depth^24,25^. Traditionally, *K*_*d*_(*λ*) is measured by the ocean color scientific community at 490 nm, and this metric is expressed as *K*_*d*_(490). To estimate *K*_*d*_(490), a neural network inversion that was trained using a combination of simulated and in situ data sets by Jamet et al.^26^ was applied in this study. The type of neural network was the Multi-Layer Perceptron. The remote sensing reflectance values (sr^−1^) at 443, 488, 531, 547, and 667 nm (R_rs_(443), R_rs_(488), R_rs_(531), R_rs_(547), and R_rs_(667)) were applied to train the neural network.

#### Model for *C*_*tsm*_ retrieval

For the retrieval of the concentration of suspended sediments (*C*_*tsm*_), the two-band ratio exponential algorithm was applied, as follows^16^:

1 $$C_{tsm} = 0.4932\exp \left( {4.2145R} \right)$$

where

2 $$R = {{R_{{rs}} \left( {645} \right)} \mathord{\left/ {\vphantom {{R_{{rs}} \left( {645} \right)} {R_{{rs}} \left( {555} \right)}}} \right. \kern-\nulldelimiterspace} {R_{{rs}} \left( {555} \right)}}$$

#### Ekman pumping

The Ekman pumping velocity was computed directly using the following equation^27,28^:

3 $${w = \frac{{1}}{\rho f}k\nabla \times \overrightarrow {\tau } }$$

where ρ is the mean density of seawater, f is the Coriolis parameter, *k* is a unit vector, and $\overrightarrow {\tau }$ is the hourly wind stress vector.

## Results and discussion

### Temporal and spatial pattern of K_d_(490)

**Figure 2:**
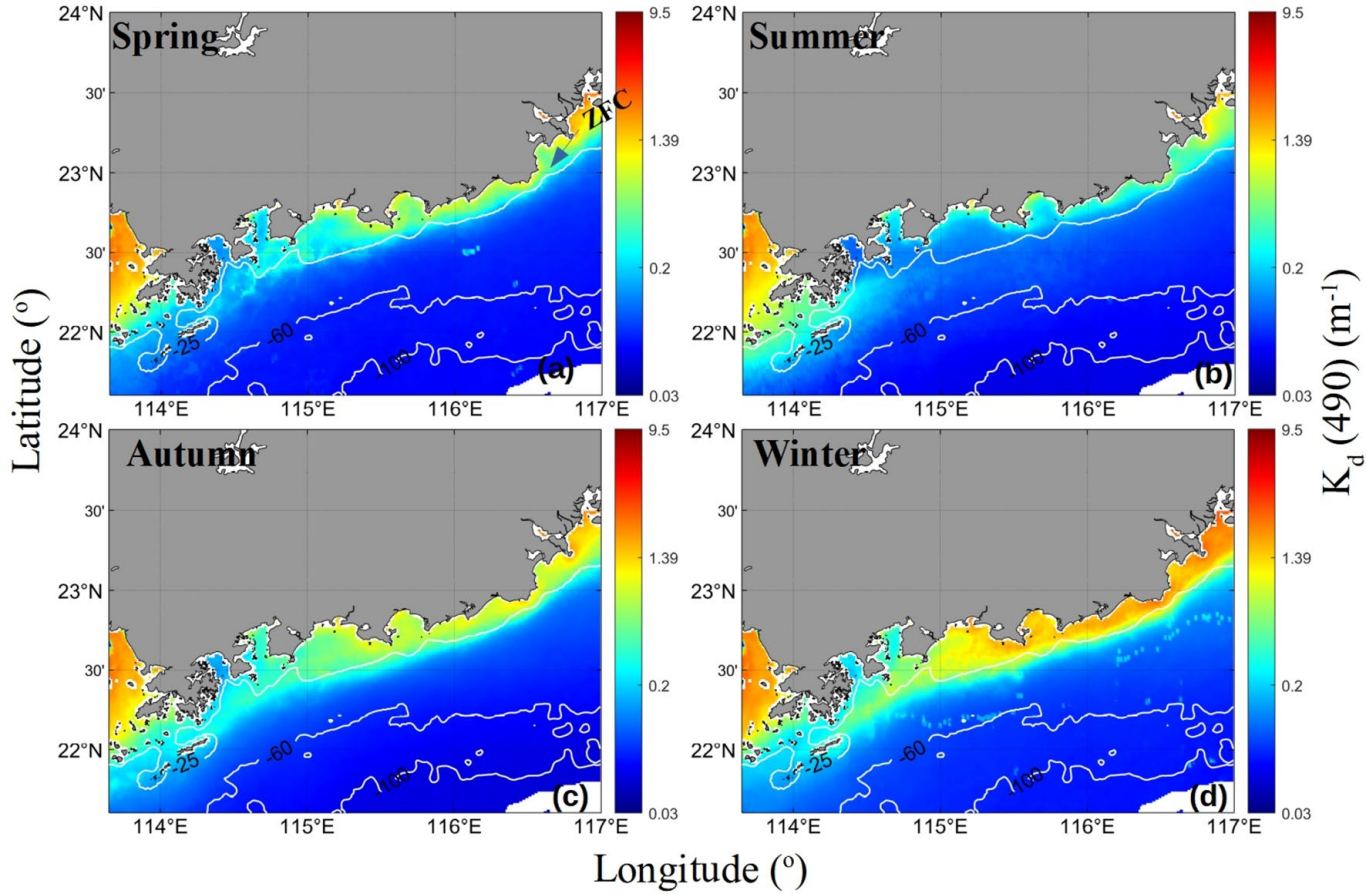
The seasonal mean *K*_*d*_(490) values (m^−1^) in four seasons from 2003 to 2017. The program R (version 3.6.3, available at https://www.r-project.org) was applied for the map.

**Figure 3:**
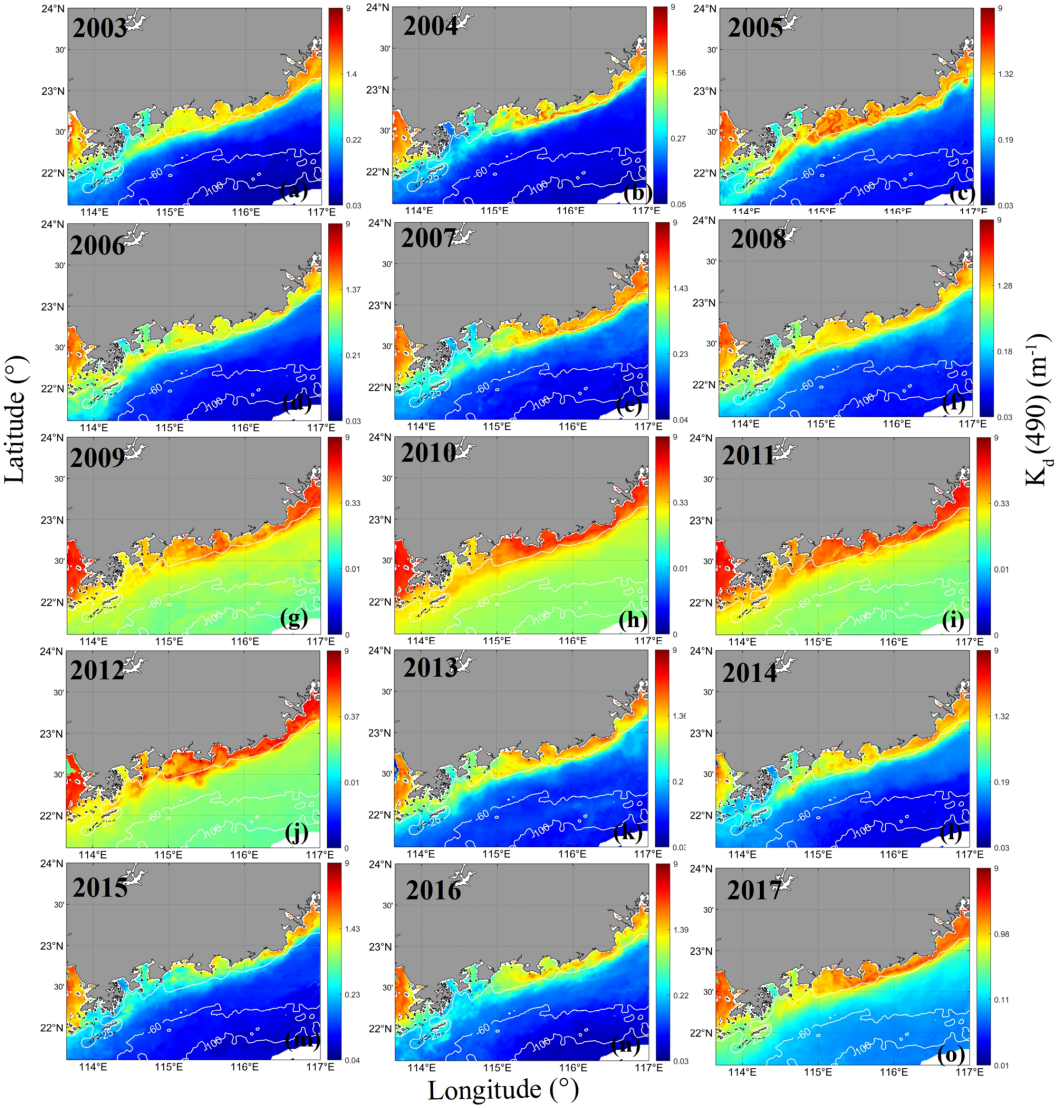
The mean *K*_*d*_(490) values (m^−1^) between November and December from 2003 to 2017.The program R (version 3.6.3, available at https://www.r-project.org) was applied for the map.

**Figure 4:**
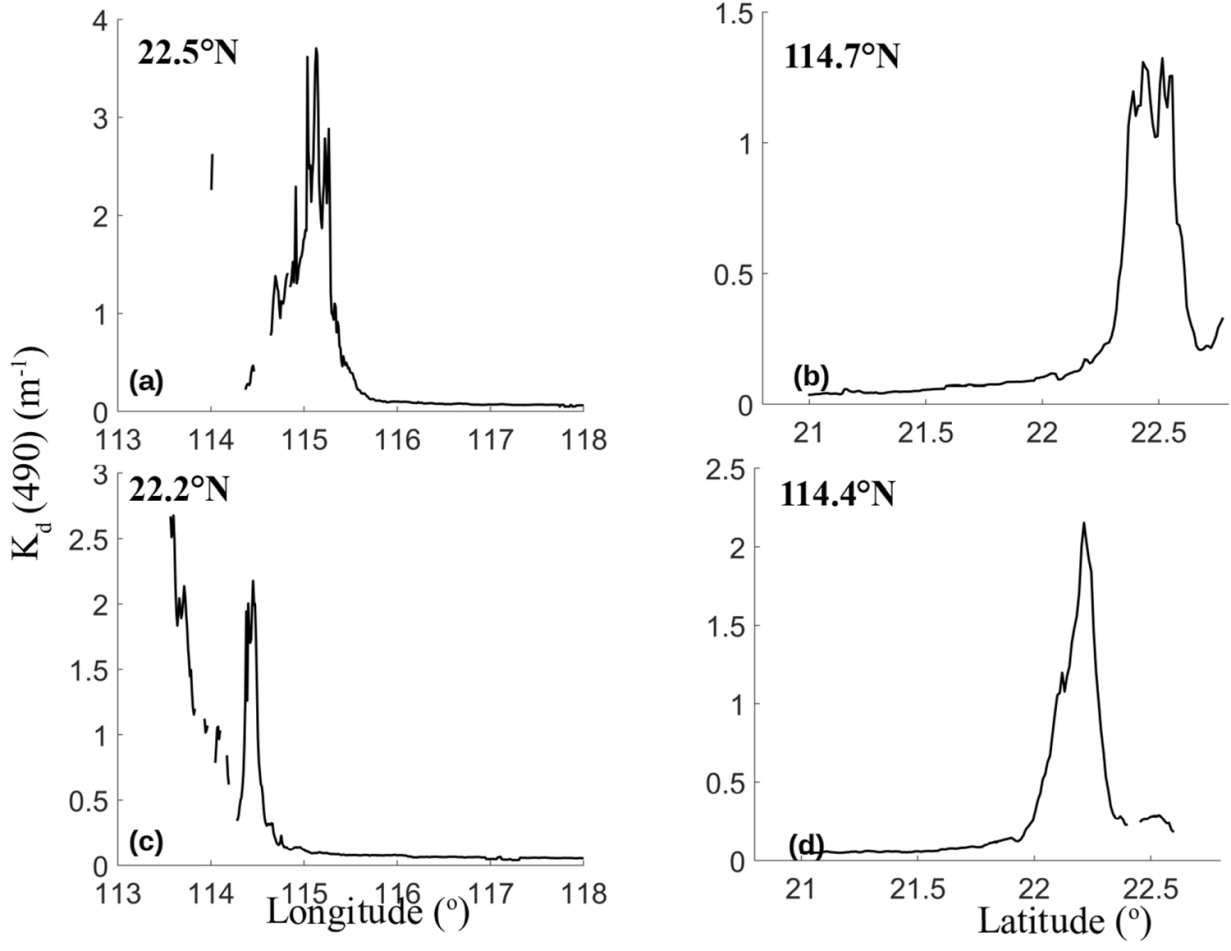
The meridional and zonal distributions of the mean *K*_*d*_(490) values in coastal waters less than 25 m in depth between November and December 2005. Panel (**a**) represents the meridional distribution of the mean *K*_*d*_(490) at 22.5°N; (**b**) represents the zonal distribution of the mean *K*_*d*_(490) at 114.7°E; (**c**) represents the meridional distribution of the mean *K*_*d*_(490) at 22.2°N; and (**d**) represents the zonal distribution of the mean *K*_*d*_(490) at 114.4°E. The program R (version 3.6.3, available at https://www.r-project.org) was applied for the map.

Figure 2 shows the mean attenuation coefficients at 490 nm (*K*_*d*_(490)) in the EGD coastal water in four seasons from 2003 to 2017, as retrieved from the MODIS data. *K*_*d*_(490) is higher in the EGD coastal water in autumn and winter than in spring and summer. In particular, a well-defined front was observed extending from the southeastern coast of Guangdong Province to the Wanshan archipelago in the SCS. Based on long-term *K*_*d*_(490) observations, the fronts were found to be prominent in November and December. The mean *K*_*d*_(490) between November and December from 2003 to 2017 is given in Fig. 3. Coastal fronts existed almost every year from 2003 to 2017. However, the lengths, directions and intensities of the fronts differed among different years. The strongest front was observed in 2005. The fronts were too weak to be distinguished in 2009. In 2005, the front was very sharp and narrow. The distributions of the mean *K*_*d*_(490) in the EGD coastal waters with depths less than 25 m between November and December 2005 are given in Fig. 4. The meridional distribution of the mean *K*_*d*_(490) values at 22.5°N and 22.2°N are given in the plots on the left, and the zonal distributions of the mean *K*_*d*_(490) values at 114.7°E and 114.4°E are given in the plots on the right. Noticeable peaks can be seen in the plots, and there is clear evidence that a significant discontinuity existed in the region. The widths of the fronts tended to decrease with decreasing latitude. The cross-frontal differences in *K*_*d*_(490) reached 3.7 m^−1^. The outer edge of the front was smooth, and the demarcation line between the lower and higher *K*_*d*_(490) values in EGD was clear and was parallel to the coastline. The front contained obvious intensity gradients. In contrast, the front was not obvious in 2009. In 2009, the edge of the front was not sharp but was slightly occluded or mottled.

### Temporal and spatial patterns of C_tsm_

**Figure 5:**
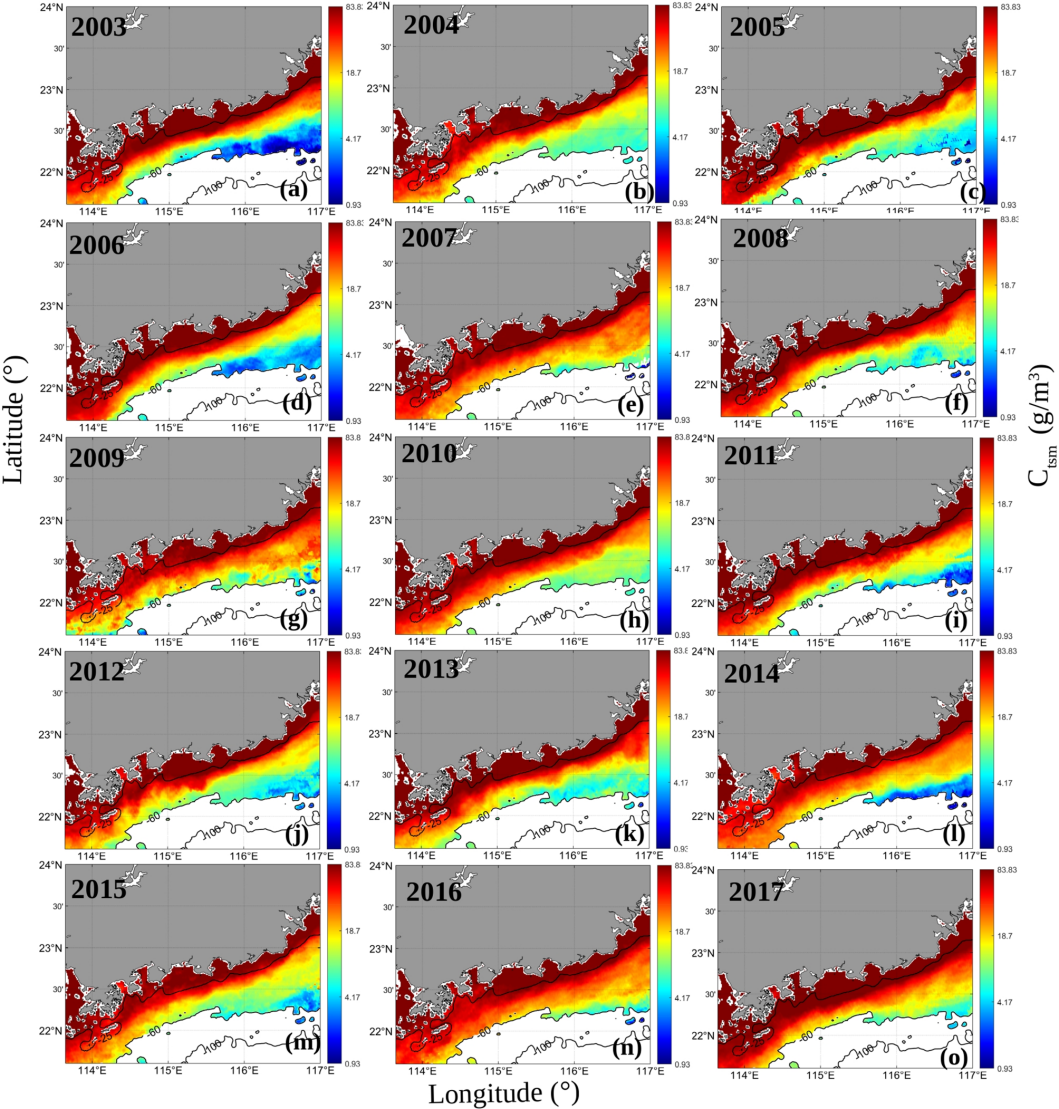
The mean C_tsm_ (g m^−3^) values between November and December from 2003 to 2017. The program R (version 3.6.3, available at https://www.r-project.org) was applied for the map.

The monthly average *C*_*tsm*_ values for the years 2003–2017 were retrieved based on remote sensing data (Fig. 5). The surface *C*_*tsm*_ was very high over the Pearl River and significantly lower over the open ocean, for which the values varied widely from 0.93 to. 83.83 g m^−3^. To track the spatial distribution and trends of *C*_*tsm*_ in coastal waters, regions with water depths greater than 25 m were masked. The *C*_*tsm*_ values were higher in the coastal waters and bounded by the bathymetric contours. The edges of the fronts almost overlapped with the bathymetric contours. The spatial distributions of the mean *C*_*tsm*_ values exhibited similar patterns with those of the *K*_*d*_(490) values. Similar to the pattern of the fronts observed in the *K*_*d*_(490) maps, the fronts seen in the *C*_*tsm*_ maps were most pronounced in 2005. The suspended sediment concentration was the dominant factor determining the *K*_*d*_(490) values in EGD coastal waters in November and December. The water transparency in this region was mainly affected by suspended sediment. *K*_*d*_(490) is an important parameter used to quantify water transparency. Consequently, a similar front can be detected in the *K*_*d*_(490) values. Therefore, all obtained data series exhibited coastal fronts extending southwestward from the southeastern coast of Guangdong Province to the Wanshan Archipelago in autumn and winter. The signature of the EGD coastal downwelling was evident in the *K*_*d*_(490) and *C*_*tsm*_ maps.

### Downwelling-favorable wind

**Figure 6:**
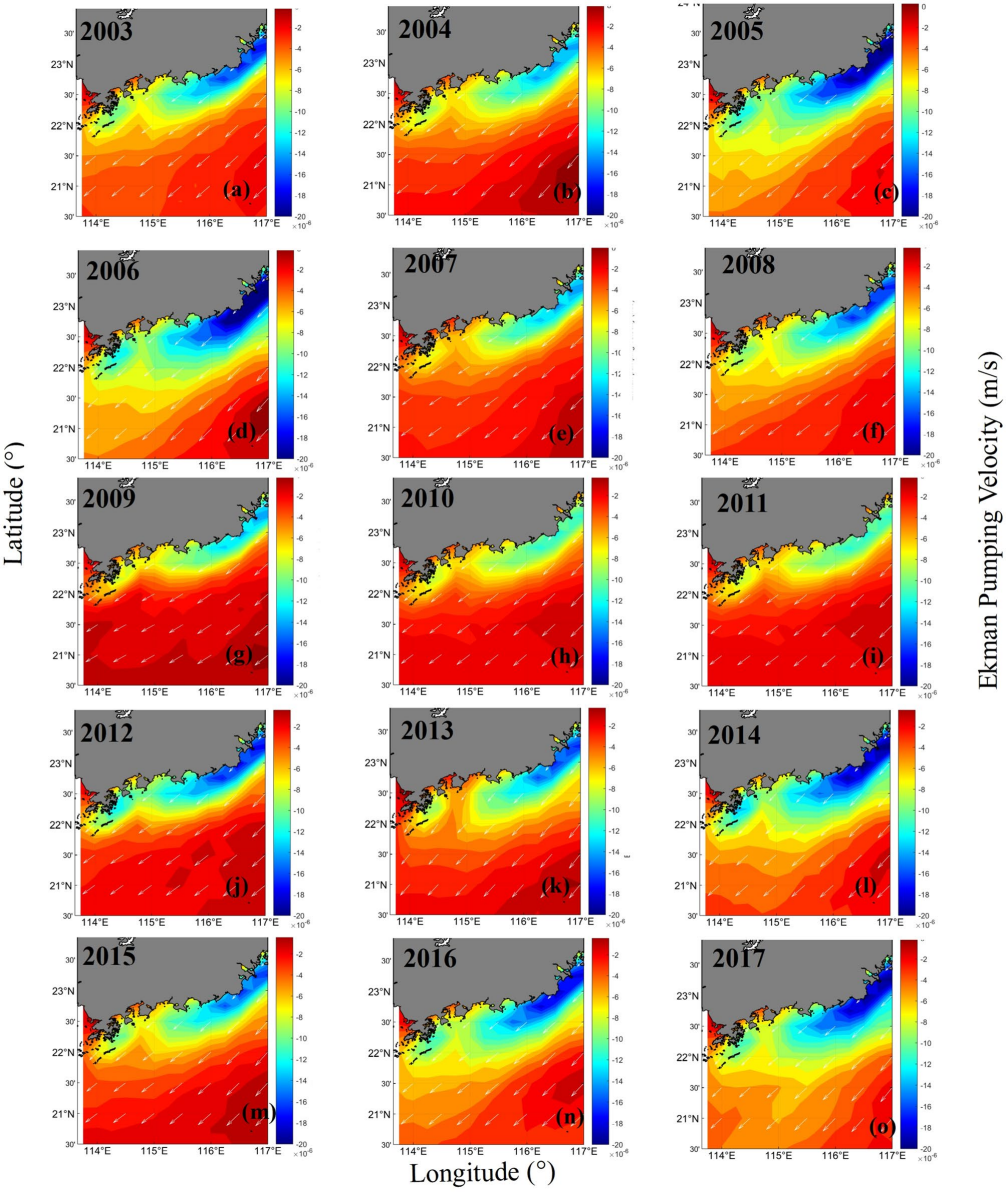
The mean Ekman pumping velocities (m s^−1^) between November and December from 2003 to 2017 (a positive value denotes upwelling, and a negative value denotes downwelling). The program R (version 3.6.3, available at https://www.r-project.org) was applied for the map.

To determine the dynamics of the coastal fronts, the monthly mean 10-m wind speeds in November and December from 2003 to 2017 were used to calculate the mean Ekman pumping velocity (Fig. 6). Figure 6 shows that a strong northeasterly monsoon wind prevailed in the NSCS. Ekman transport created onshore flows in the surface Ekman layer, and the surface waters near the shore were pushed against the shore, then piled up and sunk as a consequence of undercurrents. Vertical mixing was very strong due to the strong monsoon winds. In particular, in areas with coastal water depths of less than 25 m, *K*_*d*_(490) and *C*_*tsm*_ were higher in autumn and winter than in spring and summer. The corresponding fronts observed in the *K*_*d*_(490) and *C*_*tsm*_ series were identified in the EGD coastal waters. The sediments in the Fujian-Zhejiang coastal waters (ZFC) of the East China Sea (ECS) could be advected to the EGD coast by the strong coastal currents. For areas with depths of less than 25 m, the water is mixed well from top to bottom due to downwelling. For areas with water depths between 25 and 60 m, thermoclines and haloclines could be found at depths of 15 m in the EGD^29^. The thicknesses of these thermoclines and haloclines were found to be approximately 5 ~ 10 m. These thicknesses increased with the water depth. The thermocline intensity was approximately 0.2 °C m^−1^, and the halocline intensity was more than 0.05 m^−1^^29^. Figure 6 shows that the strength of downwelling near the Wanshan Archipelago was relatively strong in 2005 and weak in 2009. This is in accordance with the strength of the fronts observed in the *K*_*d*_(490) and *C*_*tsm*_ series.

**Figure 7:**
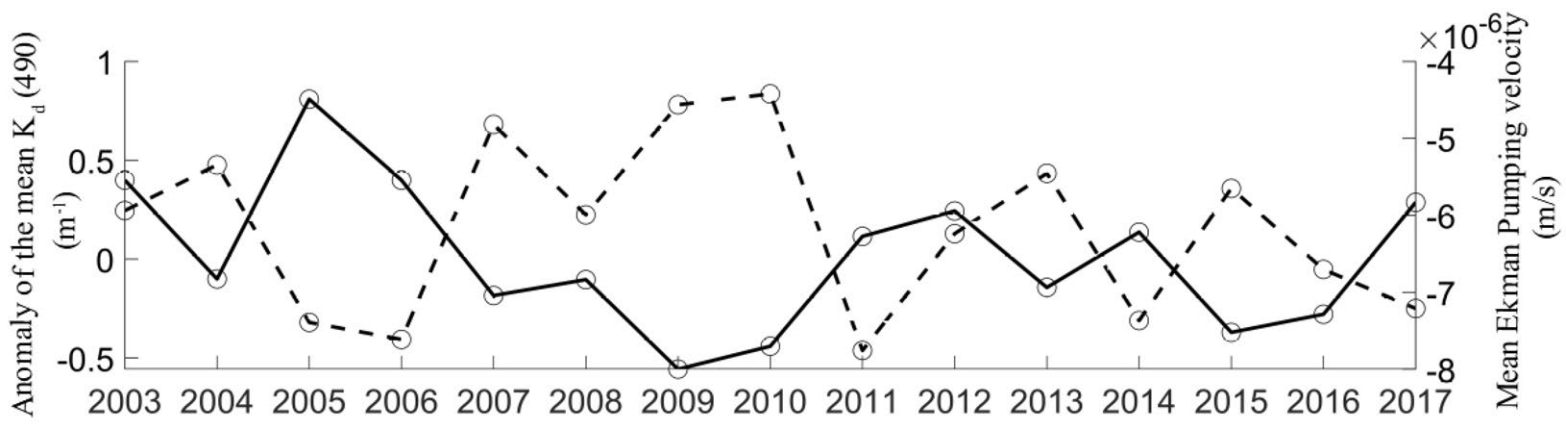
The time series of the mean *K*_*d*_(490) anomalies and the mean Ekman pumping velocities in coastal waters less than 25 m deep between November and December from 2003 to 2017. The solid line represents the mean *K*_*d*_(490) anomalies in coastal waters less than 25 m deep between November and December from 2003 to 2017. The dashed line represents the mean Ekman pumping velocities in coastal waters less than 25 m deep between November and December from 2003 to 2017. The program R (version 3.6.3, available at https://www.r-project.org) was applied for the map.

The time series of the mean *K*_*d*_(490) anomalies and the mean Ekman pumping velocities in coastal waters with depths less than 25 m between November and December from 2003 to 2017 are given in Fig. 7. The mean *K*_*d*_(490) anomalies showed an opposite trend with the mean Ekman pumping velocities. This illustrates that *K*_*d*_(490) tended to increase when strong downwelling occurred. The Pearson correlation between the monthly *K*_*d*_(490) anomalies in shallow coastal waters less than 25 m deep along the EGD coast and the monthly mean Ekman pumping velocities retrieved from the ERA5 datasets was − 0.71. Intense northeasterly winds parallel to the coast induced Ekman transport that pushed surface water to the coast. The onshore flow in the surface Ekman layer created a compensating seaward-submerged subsurface front. An overturning cross-shelf circulation induced by a downwelling jet together with breaking waves and other nonlinear wave effects triggered enhanced vertical mixing. The effects of tides, waves and downwelling on bed shear stress can cause sediment erosion in shelf seas. In addition, the shelf topography in the longshore direction is also an important factor in cross-shore bottom transport processes. Cross-shelf circulation can induce sediment resuspension when a critical bed shear stress occurs. The coastal suspended sediments were mixed well by downwelling currents and turbulence. A geostrophic coastal jet was generated when downwelling-favorable winds prevailed. The secondary circulation process transports coastal suspended sediments in the subsurface and bottom waters to the open ocean. The convective instabilities led to the development of vigorous turbulence, which induced sediment resuspension. From a remote sensing point of view, the prominent fronts observed in the *K*_*d*_(490) and *C*_*tsm*_ series can be considered evidence confirming the existence of resuspension induced by the cross-isobath transport of downwelling circulation on the shelf. The fact that the intensities of the fronts in the *K*_*d*_(490) and *C*_*tsm*_ series were clearly associated with the strength of the downwelling further illustrated this point.

**Figure 8:**
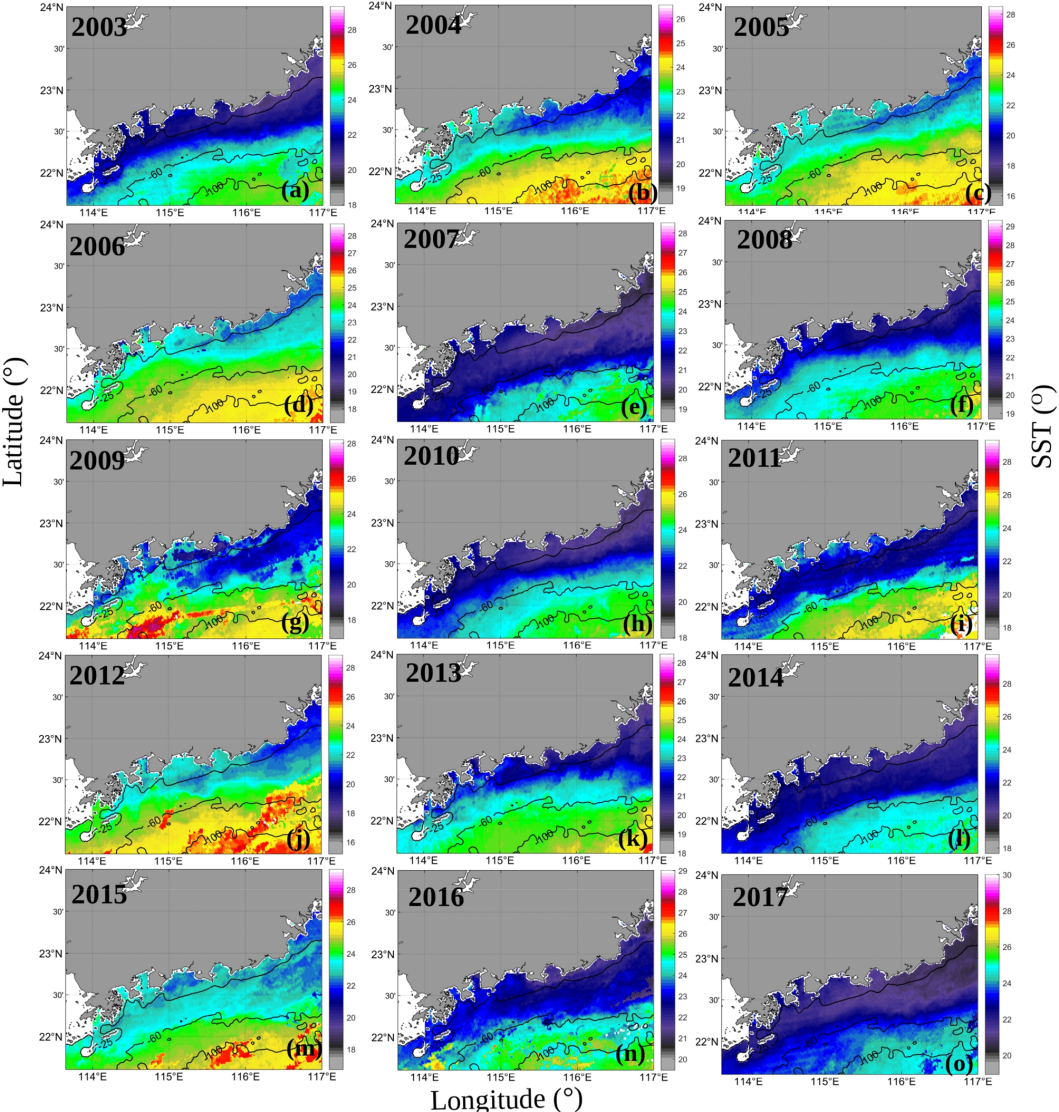
The mean SSTs between November and December from 2003 to 2017. The program R (version 3.6.3, available at https://www.r-project.org) was applied for the map.

The monthly mean SSTs between November and December from 2003 to 2017 are shown in Fig. 8. The cold currents from Fujian-Zhejiang flowed southwestward, driven by the northeast monsoon. The SSTs gradually increased from north to south along the coast of Fujian to Guangdong. The temperature gradient was very large during the occurrence of downwelling-favorable winds. The boundary between the warm and cold waters was prominent and smooth in the EGD region. The intensity of the cold tongue was relatively stronger in 2005 and weaker in 2009 from north to south (it is worth noting that the color bar varies from 15.5 to 28.5 °C in Fig. 8c). This indicates that *K*_*d*_ tended to increase as the cold water mass from the ECS increased. The nutrients and sediments in the ZFC waters could be advected southward to the EGD coast by the strong coastal currents. The lower the SST values along the EGD coast were, the more cold water was transported from the ZFC region to the EGD region.

### The fronts' development and maintenance

**Figure 9:**
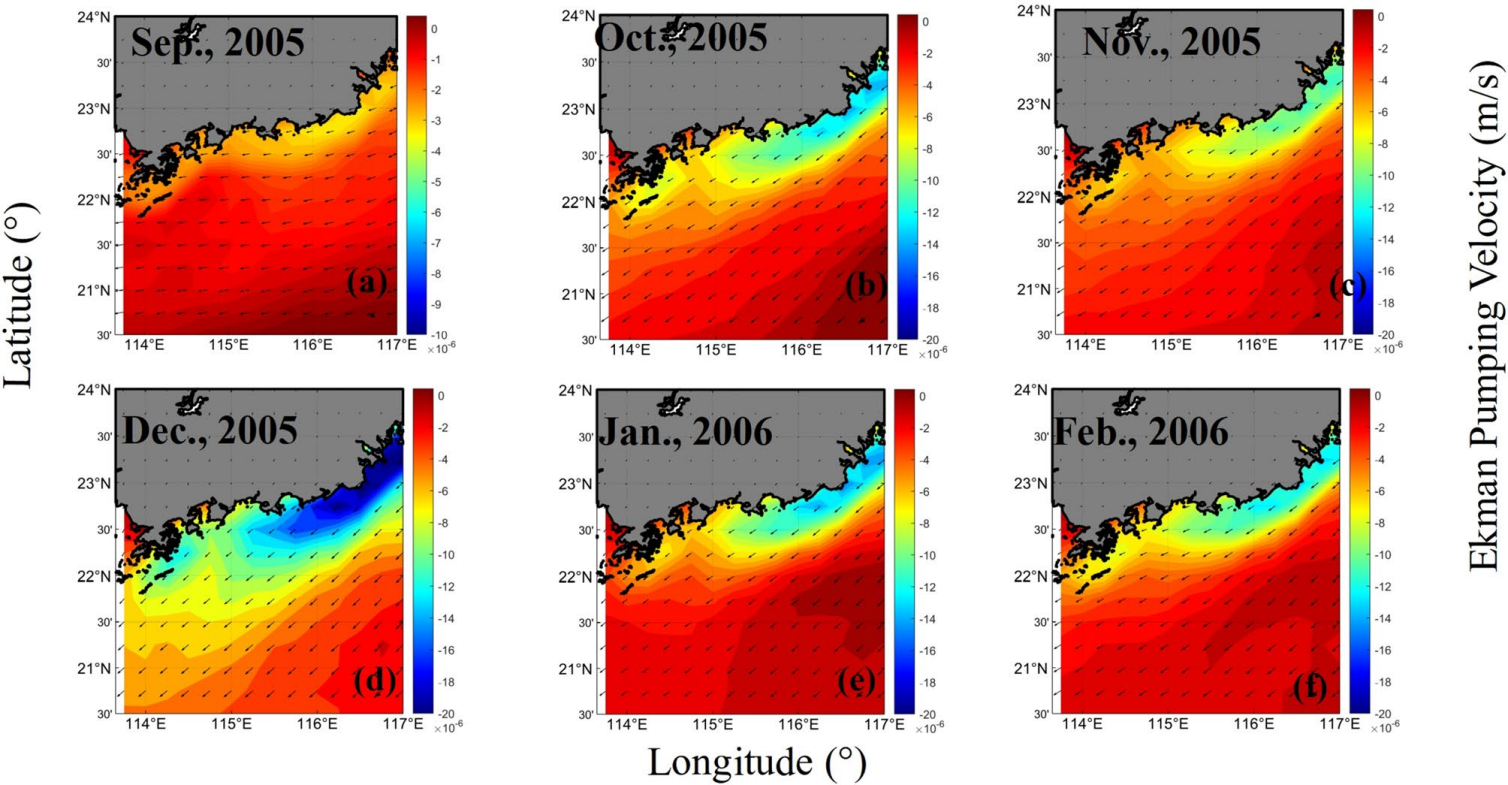
The monthly mean Ekman pumping velocities from September 2005 to February 2006 (a positive value denotes upwelling, and a negative value denotes downwelling). The program R (version 3.6.3, available at https://www.r-project.org) was applied for the map.

**Figure 10:**
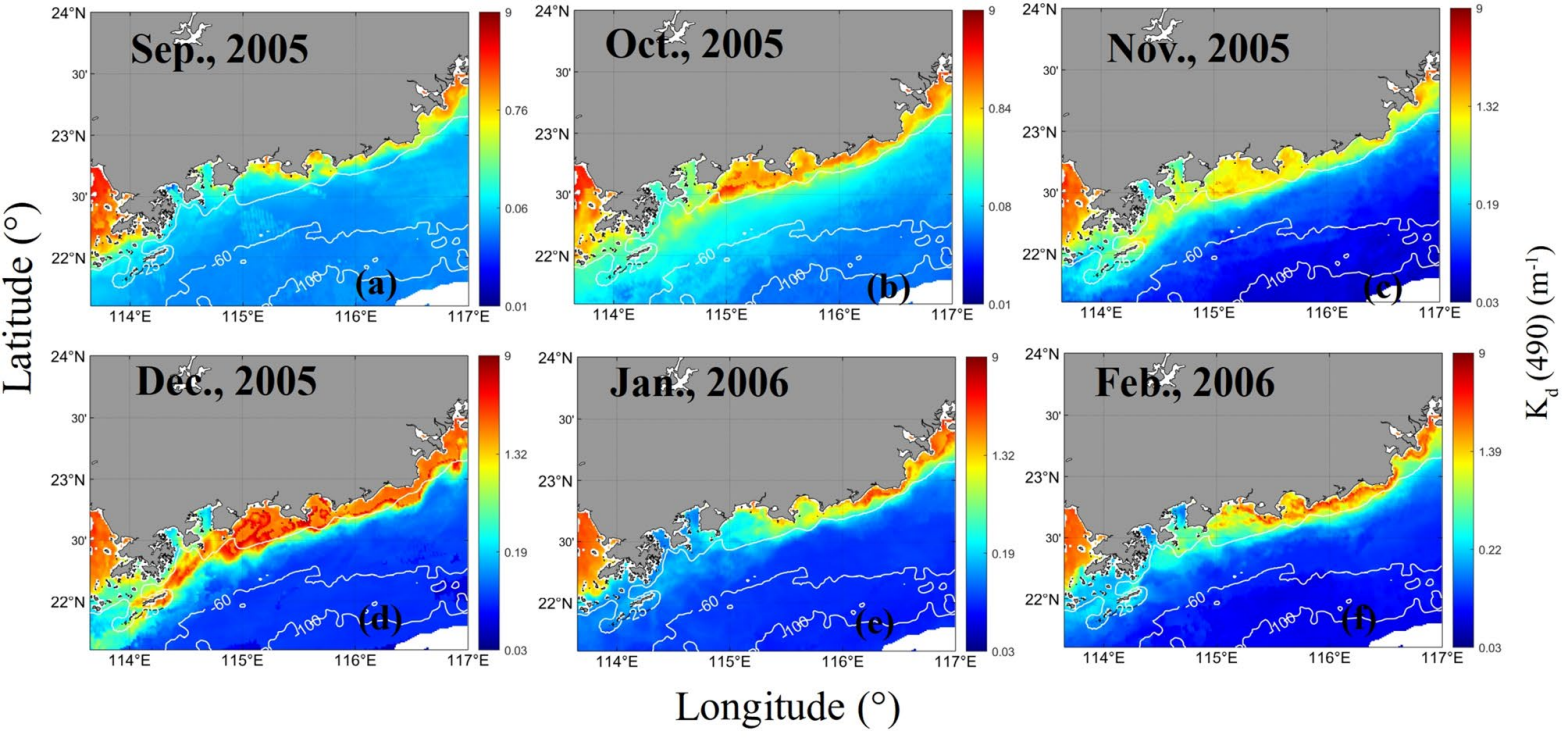
The monthly mean *K*_*d*_(490) values (m^−1^) from September 2005 to February 2006. The program R (version 3.6.3, available at https://www.r-project.org) was applied for the map.

**Figure 11:**
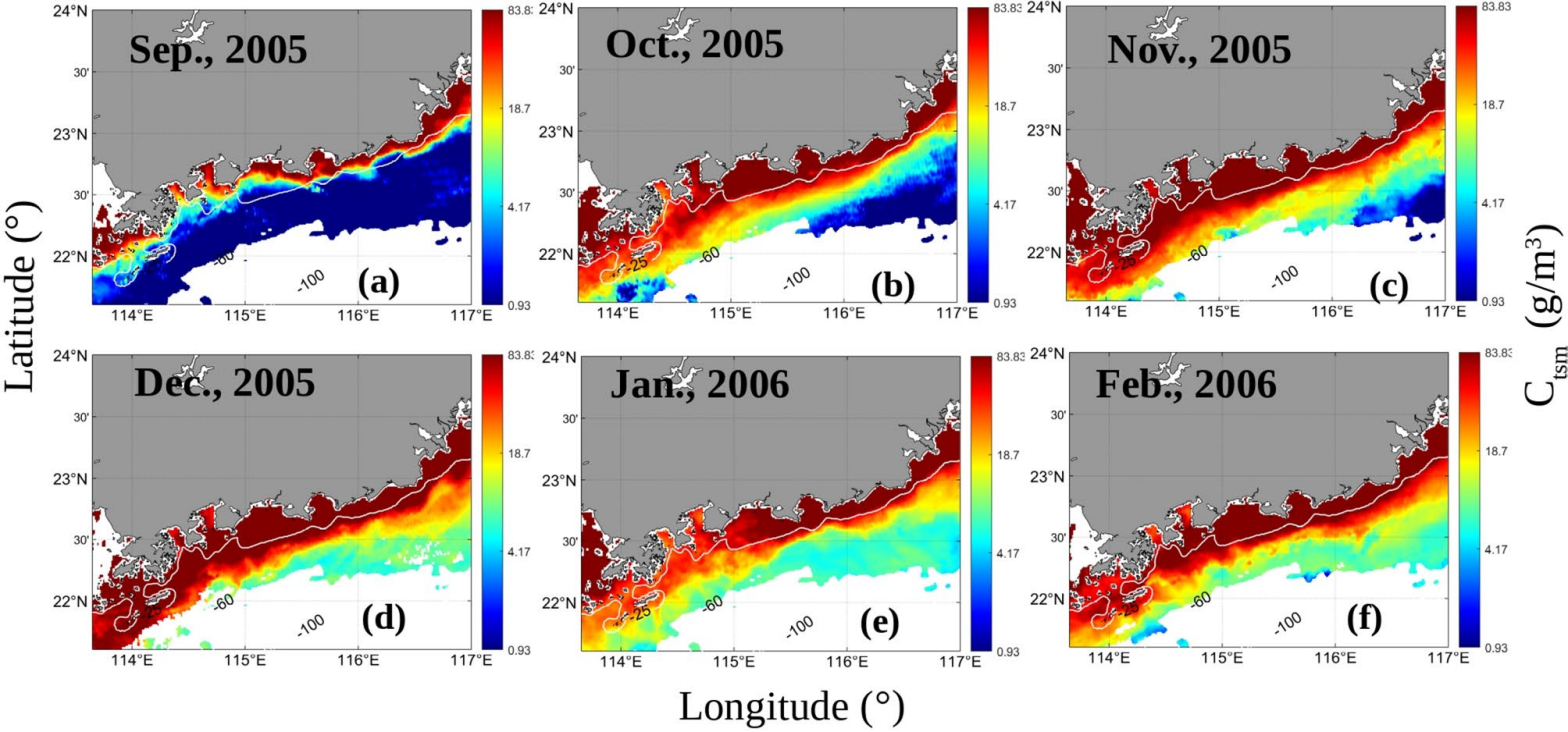
The monthly mean C_*tsm*_ (g m^−3^) values from September 2005 to February 2006. The program R (version 3.6.3, available at https://www.r-project.org) was applied for the map.

**Figure 12:**
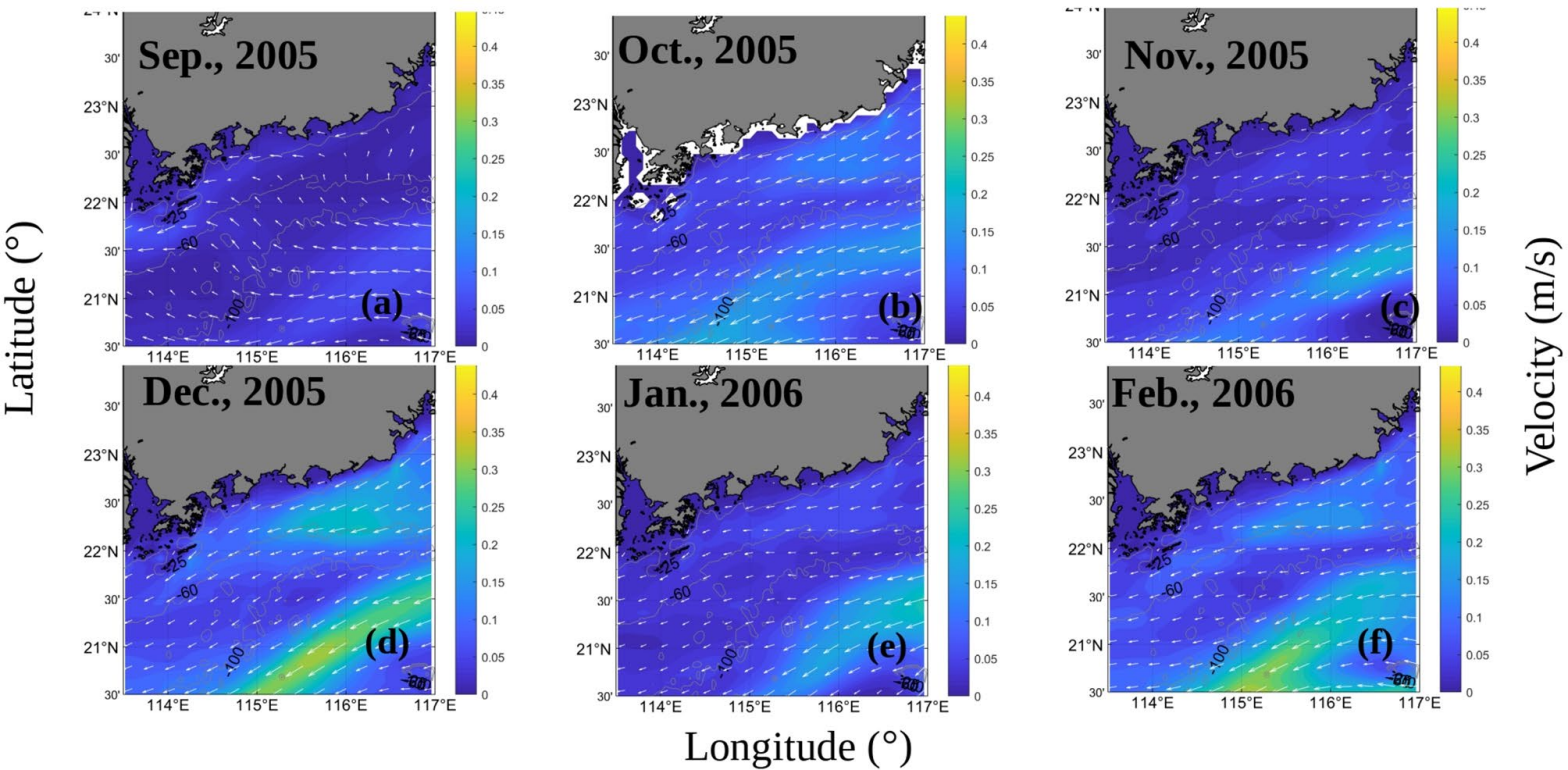
The monthly mean surface current velocities (m s^−1^) from September 2005 to February 2006. The program R (version 3.6.3, available at https://www.r-project.org) was applied for the map.

The distribution of the Ekman pumping velocities was shown to increase from September 2005 to December 2005 (Fig. 9). The corresponding fronts observed in the *K*_*d*_(490) and *C*_*tsm*_ series also exhibited similar increasing trends (Figs. 10 and 11). September and October are the autumn monsoon transitional periods over the NSCS. Figure 12 shows that the northeasterly monsoon prevailed from November 2005 to February 2006. The downwelling that occurred due to Ekman transport was most pronounced in December. The overlap between the front observed in the *K*_*d*_(490) series and the downwelling region is most pronounced. *C*_*tsm*_ is most closely associated with *K*_*d*_(490). Based on the temporal-spatial distributions of *K*_*d*_(490) and *C*_*tsm*_ from September 2005 to February 2006, fronts were not formed by September 2005. A front started to appear but was still hardly detectable in October. With the influence of downwelling-favorable winds and Guangdong coastal currents, the fronts became stronger and were most pronounced in December 2005. The fronts extended from the southeastern coast of Guangdong Province to the Wanshan Archipelago in the SCS. Then, the fronts became weaker and gradually disappeared after January 2006. The spatial distribution of *K*_*d*_(490) appeared to indicate a decrease in the offshore direction due to the nearshore sediment suspension. However, in January and February 2006, there appeared to be a location where the sediments rose to the surface offshore. This could be attributed to the intense southwestward currents that brought sediments from the northern area to this region. The monthly average global surface current velocities from the HYCOM data were calculated and are given in Fig. 12. The results showed that waters with high nutrient and sediment contents in the ZFC in the southern part of the ECS could flow into the SCS. However, the influence of the ECS coastal waters weakened from north to south. The monthly mean surface current velocities were relatively large in December 2005 and February 2006. Especially in December, a high-velocity region was observed near the EGD area. The coastal fronts were most prominent in December. Therefore, it is possible that the sediment fronts near the Wanshan Archipelago were enhanced by the resuspension that occurred in this downwelling region. The intensity of the fronts was affected by the secondary circulation induced by this downwelling region.

## Conclusion

We analyzed a long-term satellite-retrieved *K*_*d*_(490) series from 2003 to 2017. The results showed that the *K*_*d*_(490) values in EGD coastal waters were higher in autumn and winter than in spring and summer. In addition, coastal fronts could be detected in late autumn and early winter. Similar to the *K*_*d*_(490) series, the *C*_*tsm*_ series also exhibited a front in the same region at the same time. The EGD coastal downwelling signature was evident in the *K*_*d*_(490) and *C*_*tsm*_ maps. Coastal fronts existed almost every year from 2003 to 2017. The coastal fronts started in October and became weaker and gradually disappeared after January. The strongest front was observed in December of 2005. In 2005, the front was very sharp and narrow. By examining the spatial and temporal variabilities in the meridional distribution of the mean *K*_*d*_(490) values at 22.5°N and 22.2°N and the zonal distribution of the mean *K*_*d*_(490) values at 114.7°E and 114.4°E, a symmetrical peak was shown in the plots. The peak tended to narrow as the latitude increased. The fronts extended southwestward from the southeastern coast of Guangdong Province to the Wanshan Archipelago in the South China Sea. The intensities of the fronts were found to be highly associated with the downwelling intensity. The Pearson correlation between the monthly mean *K*_*d*_(490) anomalies in shallow coastal waters less than 25 m deep along the EGD coast and the monthly mean Ekman pumping velocities retrieved from the ERA5 datasets was − 0.71. The fact that the intensities of the fronts in the *K*_*d*_(490) and *C*_*tsm*_ series were clearly associated with the downwelling strength illustrated this point. The prominent fronts in the *K*_*d*_ (490) and *C*_*tsm*_ series can be considered evidence confirming the existence of resuspension induced by the cross-isobath transport of downwelling circulation on the shelf. The monthly average surface coastal current velocities estimated using HYCOM were large in February 2006, at which time the coastal fronts had already completely vanished. The sediment fronts near the Wanshan Archipelago were enhanced by the resuspension that occurred in this downwelling region. The intensity of the fronts was affected by the secondary circulation induced by this downwelling region. The direction and length of the fronts were largely determined by coastal currents. The resuspension that occurred in the downwelling region was proven by long-term satellite data analysis conducted in this study.
